# The Big Five and dark triad personality traits as predictors of emotional labour

**DOI:** 10.1080/00049530.2024.2389842

**Published:** 2024-08-12

**Authors:** Emrah Özsoy, Mark D. Griffiths, Zeynep Ak Şahinoğlu

**Affiliations:** a Sakarya Business School, Sakarya University, Sakarya, Turkey; b International Gaming Research Unit, Psychology Department, Nottingham Trent University, Nottingham, UK; c Graduate School of Business, Sakarya University, Sakarya, Turkey

**Keywords:** Emotional labour, Big Five, narcissism, Machiavellianism, psychopathy

## Abstract

**Objective:**

Emotional labour is expected, especially from service sector employees, to ensure customer satisfaction and meet corporate expectations. Previous studies have mainly focused on the effects of emotional labour on employees. Few studies have examined its predictors in terms of personality traits. In the present study, the predictive levels of the Big Five and dark triad traits on emotional labour were examined possibly for the first time.

**Method:**

Four hundred and seventy-three service sector employees participated in the research. Data were collected using an online survey comprising questions relating to the Big Five personality traits, dark triad personality traits, emotional labour and personal (demographic) information. Descriptive statistics, internal consistency test, Pearson’s correlation and hierarchical regression were used to analyse the data.

**Results:**

According to the findings, Machiavellianism was the strongest predictor of surface acting, and agreeableness was the strongest predictor of deep acting and naturally felt emotions. Narcissism was found to be a predictor of surface acting.

**Conclusions:**

As a result of examining the Big Five and dark triad traits in predicting emotional labour styles, the fact that agreeableness predicted deep acting and naturally felt emotions suggests that agreeableness should be given closer attention in recruitment and promotion decisions, especially in the service sector.

## Key Points

**What is already known about this topic**:

(1) There is a relationship between personality traits and emotional labour, but research on this subject is limited.

(2) The relationship between the Big Five, the dark triad and emotional labour has been examined separately in previous studies. However, possibly for the first time in the present study, the levels of predicting emotional labour of both personality classifications have been examined.

(3) It is known that people with dark personality traits behave opportunistically in working life and have the potential to act in line with their interests.

**What this topic adds**:

(1) Agreeableness most strongly predicted deep acting and naturally felt emotions, which are types of emotional labour that mainly lead to positive outcomes for both organizations and employees.

(2) Machiavellianism has been identified as the strongest predictor of surface acting.

(3) When the dark triad and the Big Five are taken together, it has been determined that agreeableness is the most appropriate personality trait regarding emotional labour.

## Introduction

The efforts of service sector employees are needed in many areas of daily life. Many individuals expect to interact positively with service sector employees when receiving services. Customers expect employees to exhibit behavioural patterns appropriate to the context at many stages (Nixon, 2009). Individuals are usually disturbed by the uncaring and rude attitudes of the employees. Emotional labour is the process by which service sector employees exhibit behavioural patterns and reactions appropriate to the context to ensure customer satisfaction, meet corporate and customer expectations and adapt their emotions in line with the requirements of the context (Hochschild, 1983).

Emotional labour is the product of an effort made by employees to regulate and contextualize their emotions in line with their work roles and institutional expectations. Employees obtain financial outputs in return for their efforts, which is why the concept of labour is used (Hochschild, 1983). Emotional labour has mainly been studied under three dimensions: surface acting, deep acting and naturally felt emotions (Diefendorff et al., 2005).

### Emotional labour types

Surface acting is superficially displaying emotions required by the job role while interacting with customers, even if employees are actually experiencing different emotions internally (Hochschild, 1983). Employees try to reflect their emotions through gestures, facial expressions and non-verbal communication channels to the requirements of the context. However, instead of experiencing these emotions internally, they superficially reflect their emotions (Austin et al., 2008). Although surface acting mainly requires behaviours such as making a fake smile and positive body language, in some cases, acting by the context can also occur by pretending to feel sorry for someone else (Özsoy, 2020). Because surface acting is , essentially, an insincere role-playing situation, employees display fake emotions even though they feel differently in their inner lives, which in the long term is likely to negatively affect employees’ burnout (Kılıçarslan & Özsoy, 2024) and well-being (Kayar & Aslan, 2023).

Deep acting is when employees reflect the emotions they need to reflect in line with the job’s requirements as if they were actually experiencing it. For this reason, deep acting requires more cognitive effort (Ashforth & Humphrey, 1993; Hochschild, 1983). Here, instead of reflecting fake emotions, employees display more heartfelt emotions and behaviours with customers by thinking of some of their good memories or making intense cognitive efforts (Özsoy, 2020). Employees can achieve more positive outcomes in deep acting than surface acting (Shulei & Miner, 2006).

Naturally felt emotions include spontaneous and genuine emotions, as well as behaviours that employees naturally exhibit in their inner lives, by the context, without acting superficially (Ashforth & Humphrey, 1993; Yang et al., 2019). In other words, it refers to the situation where employees already experience the emotions required by their job role while interacting with customers (Özsoy, 2020). Consequently, naturally felt emotions mainly positively affect employees’ attitudes towards work (Kılıçarslan & Özsoy, 2024). These three types differ in terms of the display of emotional labour, and the nature of each is distinct from the others (Yang et al., 2019). Surface acting can provide gains that align with customer expectations in mostly short-term interactions. Deep acting may be more important in longer-term recurring service purchases and critical situations (such as healthcare staff–patient relationships). In practice, although naturally felt emotions may not always be exhibited by service sector employees when exhibited, they provide positive results for customers (Özsoy, 2020).

In previous research, surface acting and deep acting have often been found to be negatively associated (Brotheridge & Lee, 2003; Hülsheger & Schewe, 2011), while a consistent negative relationship has also been observed between surface acting and naturally felt emotions (Bakker & Heuven, 2006; Diefendorff et al., 2005; Gabriel et al., 2015). Positive associations have also been found between deep acting and naturally felt emotions (Bakker & Heuven, 2006; Gabriel et al., 2015). In relation to employees, surface acting plays a role in reducing employees’ job satisfaction (Wen et al., 2019) and performance (Lin & Chang, 2015) increasing burnout (Kılıçarslan & Özsoy, 2024; Kim, 2020), work-family life conflict (Cheung et al., 2018) and stress (Cheung et al., 2018). On the other hand, deep acting and naturally felt emotions play a role in increasing employees job satisfaction (Cheung et al., 2018), and reducing their stress (Cheung et al., 2018) and burnout (Kılıçarslan & Özsoy, 2024; Kim, 2020).

Emotional labour has become an essential subject of study, especially in organizational psychology and management research. Employers and leaders, especially in the service sector, expect their employees to engage in emotional labour strategies and think about how they can improve their employees’ behaviours in terms of engaging more emotional labour (Özsoy, 2020). Researchers generally address the issue from the employee perspective and examine the impacts of emotional labour on employees’ attitudes and behaviours towards work and the organization (Kinman et al., 2011; van Dijk & Brown, 2006), their performance (Holman et al., 2008) and their well-being (Karimi et al., 2014).

### Personality traits and emotional labour

One of the most minor known issues about emotional labour is who is more likely to exhibit emotional labour (Kılıçarslan & Özsoy, 2024). Employers or managers may have difficulty understanding the differences in employees’ ability to exhibit emotional labour when not viewed from the perspective of differences in personality traits. However, personality differences are among the potential antecedents of emotional labour (Sohn & Lee, 2012; Walsh et al., 2020). Moreover, there are very few studies examining the extent to which personality traits can be evaluated within the scope of individual antecedents of emotional labour (Austin et al., 2008; Mróz & Kaleta, 2016). Consequently, making a clear inference on the personality antecedents of emotional labour is still difficult.

While personality differences can be antecedents of emotional labour, it is important to note that many dynamics influence variations in the display of emotional labour. As Payne (2009) emphasized, emotional labour is a skill-based activity. Moreover, factors such as individuals’ backgrounds, upbringing, education, communication skills and soft skills training in the workplace are also critical potential predictors. Indeed, it has been found that an individual’s past experiences and upbringing play a role in their level of emotional labour in professional life in later years (Fairchild & Mikuska, 2021). However, the present study focuses solely on the antecedents of emotional labour in the context of personality traits.

Examining emotional labour through the lens of personality traits is essential because it provides insight concerning the core individual differences that influence how employees manage their emotions in the workplace. By doing so, the present study offers valuable insights for human resource departments, aiding in selecting suitable candidates during the recruitment process, particularly for roles requiring emotional labour. Making informed hiring decisions in the service sector is integral to fostering economic sustainability. Therefore, the present study’s comparative analysis of various personality traits in predicting emotional labour addresses a significant gap in the existing literature and contributes to the effective employment of personnel within service sector organizations.

### The present study and hypotheses

The present study investigated the role of personality traits, specifically the Big Five and the dark triad traits, in predicting emotional labour behaviours among service sector employees. In particular, examining the Big Five together with dark triad traits, which have been found to exhibit behavioural patterns associated with adverse outcomes in organizations (Cohen & Özsoy, 2021), may provide an interesting perspective on the role of personality differences in emotional labour. The relationship between the Big Five and emotional labour has been examined in several studies, but the relationship between the dark triad and emotional labour has been inadequately examined (Walsh et al., 2020). Moreover, to the best of the present authors’ knowledge, no previous studies have examined the Big Five and dark triad traits simultaneously. Before examining the extent to which the Big Five and dark triad traits are predictors of emotional labour, the present study’s research hypotheses were constructed based on the relevant personality traits and the theoretical and empirical background of emotional labour.

When considered in terms of openness to experience, the typical characteristics of the individuals who have a high level of openness to experience are that they are open to innovations, open-minded, curious, have a low level of resistance to change and prioritize new experiences rather than conservatism (Akçay & Tuna, 2021; Benet-Martinez & John, 1998; Somer et al., 2002). Considering these basic characteristics, it can be expected that individuals with a high level of openness to experience have the potential to establish sincere relationships with individuals and have a higher tendency to behave sincerely. This may form the basis for a negative association between surface acting, which involves displaying fake and superficial emotions, and a positive association with deep acting, which involves attempts to reflect sincere emotions. In addition, individuals with high levels of openness to experience can be expected to have a positive relationship with naturally felt emotions, because they could be more likely to be more curious and comfortable in interacting with new individuals while performing their roles in the service sector. Therefore, it was hypothesized that openness to experience would be negatively associated with surface acting and positively associated with deep acting and naturally felt emotions (H_1_).

Individuals with high level of conscientiousness are success-oriented, responsible and self-disciplined (Akçay & Tuna, 2021; Benet-Martinez & John, 1998). Individuals with these characteristics can be expected to fulfil their job roles in accordance with organizational expectations. Because these individuals are success-oriented, more conscientious individuals are more likely to make the necessary effort for themselves and their organizations. In this regard, more conscientious individuals are expected to exhibit behaviours appropriate to customer expectations and context. In this respect, conscientiousness may be an essential predictor of deep acting, and naturally felt emotions. On the other hand, surface acting involves a more superficial effort. Individuals with high conscientiousness level are more likely to focus on their work, be disciplined, and have a greater tendency to do what is required for their job. In this respect, the probability of exhibiting surface acting is expected to be low. Therefore, it was hypothesized that conscientiousness would be negatively associated with surface acting and positively associated with deep acting and naturally felt emotions (H_2_).

Individuals with high level of extraversion can easily interact with others, have high energy, are social, and adapt quickly to social environments (Benet-Martinez & John, 1998; Saltürk, 2008; Somer & Goldberg, 1999). These characteristics provide the basis for individuals to behave cheerfully, energetically, and in a way that will leave a positive impression, especially in customer interaction in the service sector. Therefore, it is expected that individuals with high levels of extraversion tend to exhibit all components of emotional labour. The findings obtained in previous studies align with this expectation to some extent (Diefendorff et al., 2005; Eighie et al., 2012). Therefore, it was hypothesized that extraversion would be positively associated with surface acting, deep acting and naturally felt emotions (H_3_).

Individuals with high agreeableness are sincere, empathetic, understanding and cooperative (Cobb-Clark & Schurer, 2012; Rammstedt, 2007; Roccas et al., 2002). These characteristics increase the likelihood that employees with a high level of agreeableness will present a patient with an understanding profile in their customer interactions. Because agreeable individuals tend to be conciliatory and empathetic, they are expected to be more likely to exhibit surface and deep acting behaviours and naturally felt emotions to meet organizational and customer expectations. Partly supporting this assumption, previous studies have found positive relationships between deep acting and agreeableness (Aslan & Arı, 2018; Diefendorff et al., 2005). Therefore, it was hypothesized that agreeableness would be positively associated with surface acting, deep acting and naturally felt emotions (H_4_).

Individuals with high levels of neuroticism are emotionally unstable, whose moods can change rapidly, tend to experience negative emotions, are anxious and pessimistic, and have difficulties in the face of minor problems (Özsoy et al., 2014; Widiger & Smith, 2008). Considering these characteristics, individuals with high levels of neuroticism are expected to have a low tendency to exhibit emotional labour. In the service sector, it is possible to interact with many customers with different personality traits throughout the day, which may cause communication problems. In this respect, an individual’s ability to be emotionally stable and to keep their emotions under control is essential in order to engage in emotional labour. Consequently, it is expected that individuals with high neuroticism tendencies will be negatively associated with all emotional labour components. Therefore, it was hypothesized that neuroticism would be negatively associated with surface acting, deep acting and naturally felt emotions (H_5_).

Individuals with a high tendency towards narcissism are power and success-oriented, arrogant, consider themselves superior to others, and tend to create a good impression and establish authority in the eyes of others (Furnham et al., 2013; Jones & Paulhus, 2014; Özsoy & Ardıç, 2017). In working life, narcissists are likely to engage in emotional labour in organizations, primarily because of their high desire to gain power and create a good impression, because this can provide a basis for both being perceived positively by decision-makers and receiving positive feedback from customers in their interactions with customers. In particular, surface acting is a form of emotional labour that would be positively associated with narcissistic patterns since narcissists tend to stand in the forefront. However, individuals with a high tendency towards narcissism have tendencies such as having difficulty in establishing empathy (Burgmer et al., 2021) and emotional inconsistency (Ardıç & Özsoy, 2016). Consequently, narcissism is expected to be negatively associated with deep acting and naturally felt emotions. Therefore, it was hypothesized that narcissism would be positively associated with surface acting and negatively associated with deep acting and naturally felt emotions (H_6_).

Individuals with a high tendency towards Machiavellianism compromise moral values when necessary to achieve their interests, use flattery and tactics in human relations, and tend to act politically (Jonason & Webster, 2010; Özsoy & Ardıç, 2017). In this respect, Machiavellians have patterns that are likely to exhibit intense surface acting, which is expected from employees, especially in the service sector, in order to maintain their place in organizations and get promoted. In general, individuals with a high tendency towards Machiavellianism having difficulty in deepening relationships reduces the possibility of deep acting and naturally felt emotions. Therefore, it was hypothesized that Machiavellianism would be positively associated with surface acting and negatively associated with deep acting and naturally felt emotions (H_7_).

Finally, individuals with high levels of psychopathy are selfish and impulsive, have a lack of developed sense of compassion, and think about their pleasure rather than others (Babiak et al., 2007; Özsoy & Ardıç, 2017). In this respect, these individuals have a significant weakness in having empathy (Mullins-Nelson et al., 2006). These features potentially reduce the likelihood of these individuals exhibiting deep acting and naturally felt emotions. On the other hand, they may exhibit surface acting due to their self-interested tendencies (Özsoy, 2017) to seize opportunities in the workplace by gaining good impressions by applying surface acting. Therefore, it was hypothesized that psychopathy would be positively associated with surface acting and negatively associated with deep acting and naturally felt emotions (H_8_).

## Methods

### Participants and procedure

All participants were service sector employees, and the sample comprised 473 Turkish participants. Of these, 62.4% were women, 63.6% were married, 66.2% were private sector employees, 66.1% were white-collar employees and 29.6% had a managerial role. The sample had a mean age of 34.36 years (SD = 8.83; range = 18–72 years). With regard to education, 1.9% had primary education, 12.3% had high school and equivalent education, 65.8% had a university education, 16.1% had a master’s degree and 4% had a PhD. In the organizational hierarchy, 15.83% worked at the lowest level, 62.54% worked at the middle level and 21.54% worked at the upper level.

Data were collected using an online survey comprising questions relating to the Big Five personality traits, dark triad personality traits, emotional labour and personal (demographic) information. The survey was distributed online to employees actively working in various service sectors through the social networks of the first and third authors. The participant information form stated that only service sector employees could participate in the research. Participants who declared they did not work in the service sector were excluded. In addition, although 513 individuals participated in the research, 40 participants were removed from the dataset because they completed the survey incompletely and/or carelessly. Therefore, the final sample size was 473 participants. The data were obtained voluntarily from participants irrespective of whether they worked in the public or private sector. Before data collection, informed consent was provided by all participants. Ethical approval for the study was received from the second author’s university ethical board before the recruitment of the participants, and complied with the Helsinki Declaration (Ethics approval number: E-61923333-050.99 -327,009).

### Measures

*Emotional Labour Scale (ELS)*. The Turkish version (Basım & Beğenirbaş, 2012) of the ELS Diefendorff et al., (2005) was used to assess emotional labour. The scale consists of 13 items comprising three dimensions: surface acting (six items, e.g., *“I fake the emotions I show when dealing with customers”*); deep acting (four items, e.g., *“I make an effort to actually feel the emotions that I need to display toward others”*) and naturally felt emotions (three items, e.g., *“The emotions I show customers come naturally”*). All the items are rated from 1 (never to 5 = always). Higher scores indicate a greater level of emotional labour for each dimension. See Table 1 for internal consistency scores.

**Table 1.** Descriptive statistics and internal consistency scores on the subscales of the emotional labour scale (ELS), dark triad dirty dozen scale (DTDDS) and Big Five Inventory (BFI).

Variables	Mean	Standard deviation	Cronbach’s alpha (α)
**Emotional labour (ELS)**			
Surface acting	2.47	1.07	.89
Deep acting	3.16	1.17	.90
Naturally felt emotions	3.81	0.91	.84
**Dark triad (DTDDS)**			
Narcissism	2.44	1.14	.88
Machiavellianism	1.63	0.79	.82
Psychopathy	1.62	0.73	.70
**Big Five (BFI)**			
Openness to experience	3.78	0.68	.83
Conscientiousness	3.85	0.67	.82
Extroversion	3.73	0.81	.87
Agreeableness	4.15	0.54	.72
Neuroticism	2.85	0.76	.78

*Big Five Inventory (BFI)*. The Turkish version (Sümer et al., 2005) of the BFI (Benet-Martinez & John, 1998) was used to assess the Big Five personality traits. The scale consists of 44 items comprising five factors: openness to experience (10 items, e.g., *“I am talkative”*), conscientiousness (9 items, e.g., *“I do things efficiently”*), extroversion (8 items, e.g., *“I am outgoing, sociable”*), agreeableness (9 items, e.g., *“I like to cooperate with others”*) and neuroticism (8 items, e.g., *“I get nervous easily”*). All the items are rated from 1 (*strongly disagree*) to 5 (*strongly agree*). Higher scores indicate higher level of the specific personality trait. See Table 1 for internal consistency scores.

*Dark Triad Dirty Dozen Scale (DFDDS)*. The Turkish version (Özsoy & Ardıç, 2017) of the DTDDS (Jonason & Webster, 2010) was used. The scale has 12 items assessing each of the three dark triad traits: narcissism (four items, e.g., *“I tend to seek prestige or status”*), psychopathy (four items, e.g., *“I tend to lack remorse”*) and Machiavellianism (four items, e.g., *“I tend to manipulate others to get my way”*). All items are rated from 1 (*strongly disagree*) to 5 (*strongly agree*). Higher scores indicate greater dark triad traits. See Table 1 for internal consistency scores.

### Data analysis

Descriptive statistics (means, standard deviations, frequencies and percentages), internal consistency tests (Cronbach’s alpha), Pearson’s correlation and multiple regression were used to analyse the data.

## Results

Descriptive statistics and internal consistency values are shown in Table 1. Internal consistency (Cronbach's alpha) scores for all variables were good to excellent (0.70–0.90). Correlation analysis findings are shown in Table 2, and multiple regression analyses are shown in Table 3.

**Table 2.** Correlation analysis findings.

	Emotional labour			Dark triad			Big Five					Demographics	
Variables	SA	DA	NFE	N	M	P	OE	C	E	A	NE	Age	G
Emotional labour													
Surface acting (SA)	–												
Deep acting (DA)	.24***	–											
Naturally felt emotions (NFE)	−.36***	.21***	–										
Dark triad													
Narcissism (N)	.30***	.07	−.13**	–									
Machiavellianism (M)	.37***	.03	−.21***	.52***	–								
Psychopathy (P)	.21***	−.06	−.13**	.35***	.46***	–							
Big Five													
Openness to experience (OE)	.06	.16**	.22***	.03	−.05	−.02	–						
Conscientiousness (C)	−.12**	.15**	.26***	−.16**	−.27***	−.24***	.40***	–					
Extroversion (E)	.05	.16**	.19***	.08	.03	−.03	.45***	.31***	–				
Agreeableness (A)	−.14**	.17***	.29***	−.27***	−.38***	−.40***	.30***	.40***	.23***	–			
Neuroticism (NE)	.07	−.03	−.19***	.21***	.17***	.09	−.24***	−.37***	−.35***	−.34***	–		
Age	−.15**	−.02	−.10*	−.18***	−.09	−.03	−.12**	.11*	−.07	.01	−.11*		
Gender (G)	.02	.02	−.05	−.11*	−.15**	−.17***	.01	.03	−.01	.10*	.11*	−.06	

***p < .001, **p < .01, *p < .05, Bonferroni corrected alpha = 0.000641 and it did not change the significance level of the findings, Gender was coded as 1 = males, 2 = females.

**Table 3.** Hierarchical regression analysis findings.

	Emotional labour								
	Surface acting			Deep acting			Naturally felt emotions		
Variable	Cumulative		Simultaneous	Cumulative		Simultaneous	Cumulative		Simultaneous
	R^2^ Change	F Change	β	R^2^ Change	F Change	β	R^2^ Change	F Change	β
Step 1									
Age	0.02	F(2.47) 5.38**	.08	0.01	F(2.47) 3.21*	−.08	0.00	F(2.47) 0.15	−.04
Gender			.12			−.07			−.02
Step 2									
Narcissism	0.15	F(8.46) 10.23***	.12*	0.07	F(8.46) 4.35***	.07	0.14	F(8.46) 9.00***	−.03
Machiavellianism			.28***			.07			−.11*
Psychopathy			.04			−.04			.03
Openness to experience			.07			.04			.09
Conscientiousness			−.05			.10			.11*
Extroversion			.01			.09			.07
Agreeableness			−.01			.17**			.15**
Neuroticism			−.02			.08			−.03

***p < .001, **p < .01, *p < .05, Gender was coded as 1= males, 2 = females.

### Correlation and regression analyses

Openness to experience, conscientiousness, extroversion and agreeableness were positively associated with deep acting and naturally felt emotions. Therefore, H_1_ to H_4_ regarding deep acting and naturally felt emotions were supported. Neuroticism was negatively associated only with naturally felt emotions but not deep and surface acting. Therefore, H_5_ was supported only in terms of naturally felt emotions. Openness to experience, extroversion and neuroticism were not associated with surface acting. Conscientiousness and agreeableness were negatively associated with surface acting. Therefore, none of the first five hypotheses regarding the Big Five were supported regarding surface acting. As expected, all dark triad traits were positively associated with surface acting and negatively associated with naturally felt emotions. Therefore, H_6_ to H_8_ were supported in terms of surface acting and naturally felt emotions but not for deep acting since no dark triad traits were associated with deep acting. Dark triad traits were as follows: (i) negatively correlated with conscientiousness and agreeableness and (ii) positively correlated with neuroticism. There were no correlations with psychopathy. With regard to gender, males scored higher than females on all dark triad traits (Table 2).

Hierarchical regression analysis was conducted to test the effects of dark triad and Big Five characteristics on emotional labour types. The first step of the regression consisted of age and gender. In the second step, dark triad and Big Five traits were added. The overall regression model explained 17% variance of surface acting, 8% variance of deep acting and 14% variance of naturally felt emotions. Age and gender predicted 2% variance of surface acting and 1% variance of deep acting. After controlling for age and gender, the second step explained 15% variance of surface acting, 7% variance of deep acting and 14% variance of naturally felt emotions. Only narcissism and Machiavellianism significantly predicted surface acting. Agreeableness predicted deep acting and naturally felt emotions, and Machiavellianism negatively predicted naturally felt emotions. Therefore, in terms of personality traits, Machiavellianism was the strongest predictor of surface acting, and narcissism also predicted surface acting. Among the Big Five traits, only agreeableness predicted deep acting and naturally felt emotions. Machiavellianism negatively predicted naturally felt emotions and conscientiousness positively predicted naturally felt emotions (Table 3).

## Discussion

Previous empirical studies have found that emotional labour strategies are related to employees’ personality traits (Austin et al., 2008). However, possible predictors of emotional labour in the context of personality traits have yet to be adequately examined (Kılıçarslan & Özsoy, 2024; Walsh et al., 2020). Therefore, the present study examined the extent to which the Big Five and the dark triad traits were associated with and predicted facets of emotional labour. H_1_ to H_4_ were supported regarding deep acting and naturally felt emotions but not for surface acting. H_5_ was supported only in terms of naturally felt emotions. H_6_ to H_8_ were supported in terms of surface acting and naturally felt emotions but not for deep acting.

### Interpretation of findings

In the correlation analysis, the expected relationships between the Big Five traits and emotional labour types were supported mainly in terms of deep acting and naturally felt emotions, but not for surface acting. The reason why openness to experience (H_1_) and extroversion (H_3_) traits were not associated with surface acting may be related to the fact that both personality traits have a more energetic and relaxed tendency to interact with others (Özsoy et al., 2014). These features can enable such individuals to establish closer contact with customers rather than displaying fake and contrived smiles.

Conscientiousness (H_2_) was negatively associated with surface acting, while the other Big Five components were not associated with surface acting. The negative association between conscientiousness with surface acting can be attributed to the fact that individuals with a high level of conscientiousness might try to internalize customer relationships deeply and realistically by being more empathetic rather than engaging in fake emotions because individuals with high level of conscientiousness are disciplined (Özsoy et al., 2014), adaptable (Ardıç & Özsoy, 2016), success-oriented and diligent (Çiçek & Aslan, 2020). Therefore, those traits may provide the basis for them to embrace their work roles more sincerely and better meet organizational expectations.

Agreeableness (H_4_) was not associated with surface acting. This might be because agreeable individuals are patient and get along well with others (Poropat, 2009). This may allow them to interact more realistically and sincerely with customers rather than acting superficially. The reason why openness to experience and extroversion traits are not associated with surface acting may be related to the fact that both personality traits have a more energetic and relaxed tendency to interact with others (Özsoy et al., 2014). These features can enable such individuals to establish closer contact with customers rather than displaying fake and contrived smiles.

A negative association was expected between neuroticism (H_5_) and all emotional labour types, but only naturally felt emotions and neuroticism were negatively associated. In naturally felt emotions, employees do not need to make extra effort and should be happy internally. However, the unpredictable moods of neurotic-oriented individuals can be seen as an obstacle to achieving such sustainability. Additionally, surface acting and deep acting were not associated with neuroticism, which may be related to neuroticism’s pessimistic and sad nature (Widiger & Smith, 2008).

As expected, all dark triad traits were positively associated with surface acting and negatively associated with naturally felt emotions (H_6_ to H_8_). This is an expected finding, as aforementioned (Walsh et al., 2020). However, a negative association was expected between deep acting and dark triad traits, but this was not the case. Since deep acting requires intense efforts to understand the other individuals’ emotions better and present emotions appropriate to the context (Ashforth & Humphrey, 1993), this may be due to dark personality traits’ superficial and self-oriented nature (Özsoy, 2017). Additionally, and similar to previous studies (Jonason & Webster, 2010; Özsoy, 2017), males scored higher than females on all dark personality traits. The study’s most notable finding was that Machiavellianism was the strongest predictor of surface acting, in which eight personality traits were included as independent variables. Narcissism also predicted surface acting, whereas no other personality traits did so. The strongest predictor of deep acting and naturally felt emotions was agreeableness. Additionally, Machiavellianism negatively predicted naturally felt emotions.

These findings show that Machiavellianism and narcissism are more prone to surface acting in the service sector, and agreeableness is a strong predictor of deep acting. Since deep acting provides more desired outcomes for the organization and employees (Kılıçarslan & Özsoy, 2024) compared to surface acting, agreeableness has been identified as the personality trait that potentially serves the most favourable results among the dark triad and Big Five traits within the scope of the present study. Indeed, the negative association between dark personalities and destructive and counterproductive behaviours in the workplace (Cohen & Özsoy, 2021) and agreeableness (Ardıç & Özsoy, 2016) might be interpreted as agreeable individuals having the potential to build constructive and sustainable relationships in organizations. In support of all these, a meta-analysis conducted on agreeableness (i.e., Wilmot & Ones, 2022) determined that agreeableness was positively associated with many outcomes, such as relational investment, teamwork, work investment and social integration.

Employers put great effort into hiring suitable personnel, especially in the service sector, where customer satisfaction is the key to success. Employees who engage in healthy interactions with customers ensure customer satisfaction. Therefore, employers need to select the most suitable personnel. The present study examined the factors influencing emotional labour, showing employers which personality traits can contribute more in service setting. The findings highlight that agreeableness would likely be a sought-after trait in the service sector.

### Practical implications

When the findings are evaluated in the context of working life in general, it is understood that agreeableness is a critical trait in interaction with colleagues and customers in working life. Considering that deep acting and naturally felt emotions contribute to the well-being of employees (Kayar & Aslan, 2023; Kılıçarslan & Özsoy, 2024), more attention should be paid to the agreeableness tendency in recruitment and promotion decisions. The dark triad traits are positively associated with surface acting, but surface acting can lead to employee burnout (Kılıçarslan & Özsoy, 2024). In other words, surface acting potentially forms the basis for decreased productivity in the organization and the possibility of increasing customers’ perception of insincerity in the face of surface acting (Özsoy, 2020). This indicates that surface acting is a form of emotional labour that does not provide much positive contributions in terms of its consequences compared to other emotional labour components.

The service sector is an area of intense competition, and interaction with customers in the service sector is an essential indicator of customer satisfaction (Santouridis & Veraki, 2017). Healthy communication and positive interaction with employees are among the basic expectations of customers when receiving service (Hassan et al., 2015). In this regard, service sector organizations make intense efforts to meet customer expectations in the interaction between employees and customers in order to gain a competitive advantage and ensure sustainability (Mithas et al., 2005). In this context, in order to meet the expectations of customers with many different emotions and personality traits, service sector employees are expected to behave according to the context, such as smiling, being polite and being positive. Sometimes, reactions need to be given according to the emotional state of the people interacting (Özsoy, 2020). For example, in the healthcare industry, if unpleasant information needs to be conveyed to the patient, the healthcare professional must convey this information appropriately and with empathy.

Although many factors may be influential in employees’ emotional labour, one of them (as seen in the present study) is personality traits. First, since working in the service sector requires constant emotional labour, employees should give importance to personality-job harmony when making career plans. Based on the present study’s findings, individuals with high levels of neuroticism are individuals with a low tendency to exhibit emotional labour when interacting with customers in the service sector. Here, individuals with high levels of neuroticism can gravitate towards areas that are more suitable for them according to their cognitive abilities and personal preferences.

Depending on the dynamics of the labour market, access to individuals who can be employed in the service sector may not be easy in all cases. Although this situation potentially puts employers in a difficult situation in some cases in recruitment, the present study’s findings indicate that individuals with high levels of agreeableness will be better at hiring in the service sector. In relation to the findings, especially in terms of deep acting and naturally felt emotions, recruiting individuals with high neuroticism and dark triad traits in the service sector does not appear advantageous. In this regard, it will be beneficial for organizations to consider these issues when recruiting for jobs in the service sector, where individuals interact directly with customers.

### Limitations and future research suggestions

The present study has a number of limitations. The data were self-reported, obtained from a single culture, and the sample size was modest. Moreover, possible individual factors (e.g., job stress, job commitment, job satisfaction and personal performance) and organizational factors (e.g., organizational performance, customer satisfaction and organizational efficiency) that may have influenced emotional labour were not included in the study. This limitation makes it difficult to understand the potential antecedents and consequences of emotional labour more comprehensively. In future research, the duration of working experience in the service sector, other personality traits and individual differences (e.g., positive psychology components, perfectionism and level of ambition), the individual’s social life satisfaction (e.g., relationship satisfaction, family satisfaction and social relations) and work-related outcomes, attitudes and behaviours should also be included in research models. Such potential studies could be conducted with samples from different cultures. This would allow the predictors of emotional labour to be understood more comprehensively (in micro and meso terms), and a connection could be established between the predictors and consequences.

## Conclusion

In sum, the present study found that the strongest predictor of surface acting was Machiavellianism, and the strongest predictor of deep acting and naturally felt emotions was agreeableness. The present study also examined the personality antecedents of emotional labour by considering the Big Five and the dark triad personality traits together, and found that agreeableness was a valuable and preferable trait, especially among service sector employees. The present study contributes to the existing literature in organizational psychology by highlighting the critical role of agreeableness in regulating emotional labour processes. The findings not only advance our understanding of the complex interplay between personality traits and emotional labour, but also offer practical implications for organizational practices.

## Data Availability

The dataset generated during and/or analysed during the current study is available from the corresponding author on reasonable request.
